# A blood aging clock defines disease-induced deviation from healthy molecular aging

**DOI:** 10.1093/bib/bbag605

**Published:** 2026-10-30

**Authors:** Haotian Li, Sai Liu, Zheng Zhou, Qiulun Lu, Guangji Wang, Zheying Zhu, Xinuo Li

**Affiliations:** State Key Laboratory of Natural Medicines, China Pharmaceutical University, 639 Longmian Avenue, Jiangning District, Nanjing 211198, Jiangsu, China; State Key Laboratory of Natural Medicines, China Pharmaceutical University, 639 Longmian Avenue, Jiangning District, Nanjing 211198, Jiangsu, China; Department of Computer Science, RWTH Aachen University, Ahornstr. 55, Aachen 52074, Germany; State Key Laboratory of Natural Medicines, China Pharmaceutical University, 639 Longmian Avenue, Jiangning District, Nanjing 211198, Jiangsu, China; State Key Laboratory of Natural Medicines, China Pharmaceutical University, 639 Longmian Avenue, Jiangning District, Nanjing 211198, Jiangsu, China; School of Pharmacy, The University of Nottingham, University Park, Nottingham NG7 2RD, United Kingdom; State Key Laboratory of Natural Medicines, China Pharmaceutical University, 639 Longmian Avenue, Jiangning District, Nanjing 211198, Jiangsu, China

**Keywords:** blood aging predictor, transcriptomic aging clock, age acceleration, disease burden, deep learning, systems biology

## Abstract

Aging shapes disease susceptibility and recovery, yet it remains unclear whether disease-induced molecular perturbations can be quantitatively mapped onto healthy aging trajectories. Here, we integrated 10 753 whole-blood transcriptomic profiles from 23 cohorts and developed a blood aging predictor (BAP) using 5808 healthy individuals to define a reference trajectory of blood molecular aging. Applying BAP to COVID-19 revealed marked transcriptomic age acceleration (AgeAccel), with patients showing an average AgeAccel of 9.34 years after cohort-specific baseline correction. This aging-referenced deviation occurred across age groups and was associated with interferon signaling, immune-inflammatory activation, cytokine and neutrophil-related programs, DNA damage responses, proteostasis stress, and impaired cellular quality-control pathways. Longitudinal analysis showed that AgeAccel increased during acute infection but declined during recovery, indicating a potentially reversible aging-like molecular state at the cohort level rather than irreversible aging. Extension to sepsis, influenza, and major depressive disorder further demonstrated that elevated AgeAccel captures disease-associated molecular burden across distinct pathological contexts. Together, our study redefines transcriptomic AgeAccel as an interpretable measure of disease-induced deviation from healthy molecular homeostasis and provides a quantitative framework for assessing systemic disease burden and recovery dynamics from peripheral blood transcriptomes.

## Graphical Abstract

**Graphical Abstract:**
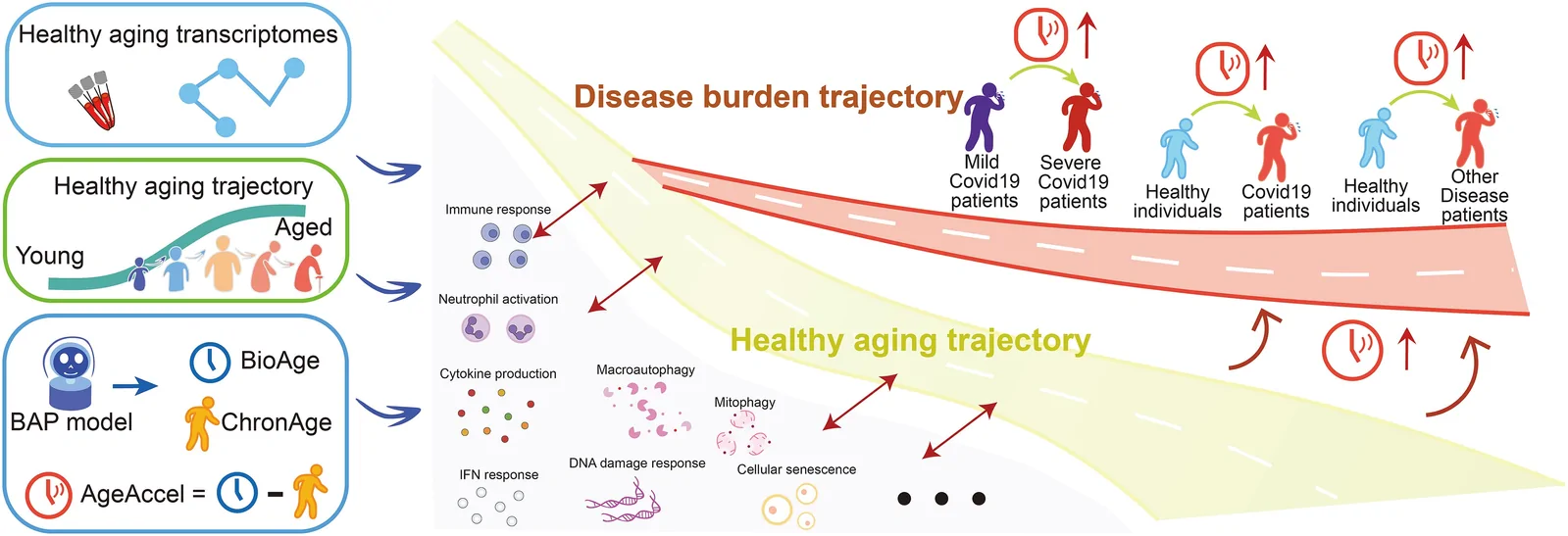

## Introduction

Aging is a major determinant of disease susceptibility, clinical resilience, and recovery capacity across a wide spectrum of chronic and acute conditions, including cardiovascular diseases, malignancies, neurodegenerative disorders, and adverse infection-related outcomes [1–3]. At the molecular level, healthy aging is accompanied by coordinated yet progressive remodeling of immune homeostasis, inflammatory tone, proteostasis, mitochondrial function, DNA damage responses, and cellular quality-control mechanisms [4, 5]. These age-associated programs not only reflect the gradual decline of physiological reserve but also provide a biologically meaningful reference trajectory against which disease-induced molecular perturbations may be assessed [1–5]. However, although aging is widely recognized as a risk factor for disease, whether and how disease states deviate from, accelerate, or transiently reshape healthy aging trajectories remains insufficiently characterized in a systematic and quantitative manner [2, 6, 7].

COVID-19 provides a particularly relevant model for addressing this question [8, 9]. Severe acute respiratory syndrome coronavirus 2 (SARS-CoV-2) infection induces robust interferon responses, cytokine release, immune-cell compositional shifts, and systemic disruption of molecular homeostasis, many of which partially overlap with biological processes implicated in aging, including inflammaging, immune dysregulation, mitochondrial stress, and impaired cellular quality control [9–11]. These features raise the possibility that acute infection may drive an aging-like molecular state in peripheral blood. Yet it remains unclear whether COVID-19-associated transcriptomic alterations can be quantitatively positioned relative to healthy aging trajectories at the individual level. Establishing an interpretable molecular reference framework based on healthy blood aging may therefore provide a powerful strategy to measure COVID-19-induced, and potentially broader disease-induced, deviations from healthy molecular homeostasis [12, 13].

Biological age clocks provide a quantitative approach to capture aging-associated molecular states and to estimate the extent to which an individual deviates from expected aging trajectories [14, 15]. Existing clocks have been developed using DNA methylation, proteomic, metabolomic, transcriptomic, and multiomics features, with age acceleration (AgeAccel) commonly defined as the difference between predicted biological age and chronological age [14–19]. However, different omics-based clocks capture distinct layers of aging biology. DNA methylation clocks, although widely used and highly stable, mainly reflect relatively persistent epigenetic remodeling [14, 15]. By contrast, the transcriptome is highly dynamic and directly reflects gene-expression programs, immune-cell states, and pathway activities, making it particularly suitable for detecting rapid molecular perturbations induced by infection, inflammation, and systemic disease [15, 17, 18]. Thus, blood transcriptomic AgeAccel may provide a sensitive and interpretable readout of disease-induced deviation from healthy molecular aging trajectories.

In this study, we integrated 10 753 whole-blood transcriptomic profiles from 23 independent datasets to systematically examine the relationship between COVID-19-associated molecular perturbations and healthy aging in peripheral blood. We first compared shared transcriptomic features between COVID-19 and aging, and then developed a blood aging predictor (BAP) using healthy individuals to establish a reference trajectory of blood molecular aging. Applying this framework to COVID-19, we found that AgeAccel captures infection-induced aging-like molecular deviation, immune-inflammatory burden, molecular disease severity, and dynamic recovery-associated changes. We further extended the framework to sepsis, influenza, and major depressive disorder, demonstrating that AgeAccel can quantify disease-associated molecular burden across distinct pathological contexts. Together, our study proposes that healthy aging trajectories can serve as a biologically meaningful reference coordinate for measuring disease-induced disruption of molecular homeostasis, systemic health deviation, and recovery dynamics.

## Results

### COVID-19 and aging share common transcriptomic signatures

To determine whether COVID-19 shares common molecular features with human aging, we first integrated nine publicly available whole-blood transcriptomic cohorts, comprising a total of 6491 samples, and established an Aging cohort (Fig. 1A, Supplementary Tables S1-S2). Following standardized preprocessing, stable gene expression filtering, and batch-effect correction using neuroCombat while preserving age-related information, a total of 11 366 genes were retained for subsequent analyses (Fig. 1B) [20]. We next performed association analysis using the limma framework with age treated as a continuous variable [21]. Using a significance threshold of false discovery rate (FDR) < 0.05, we identified 2977 genes whose expression decreased with age and 1609 genes whose expression increased with age (Supplementary Table S3). Notably, several well-established age-associated genes, including *LRRN3*, *CXCR5*, and *NRCAM*, were successfully identified (Fig. 1C), indicating that the integrated cohort reliably captures age-related transcriptomic signatures in peripheral blood [17].

**Figure 1:**
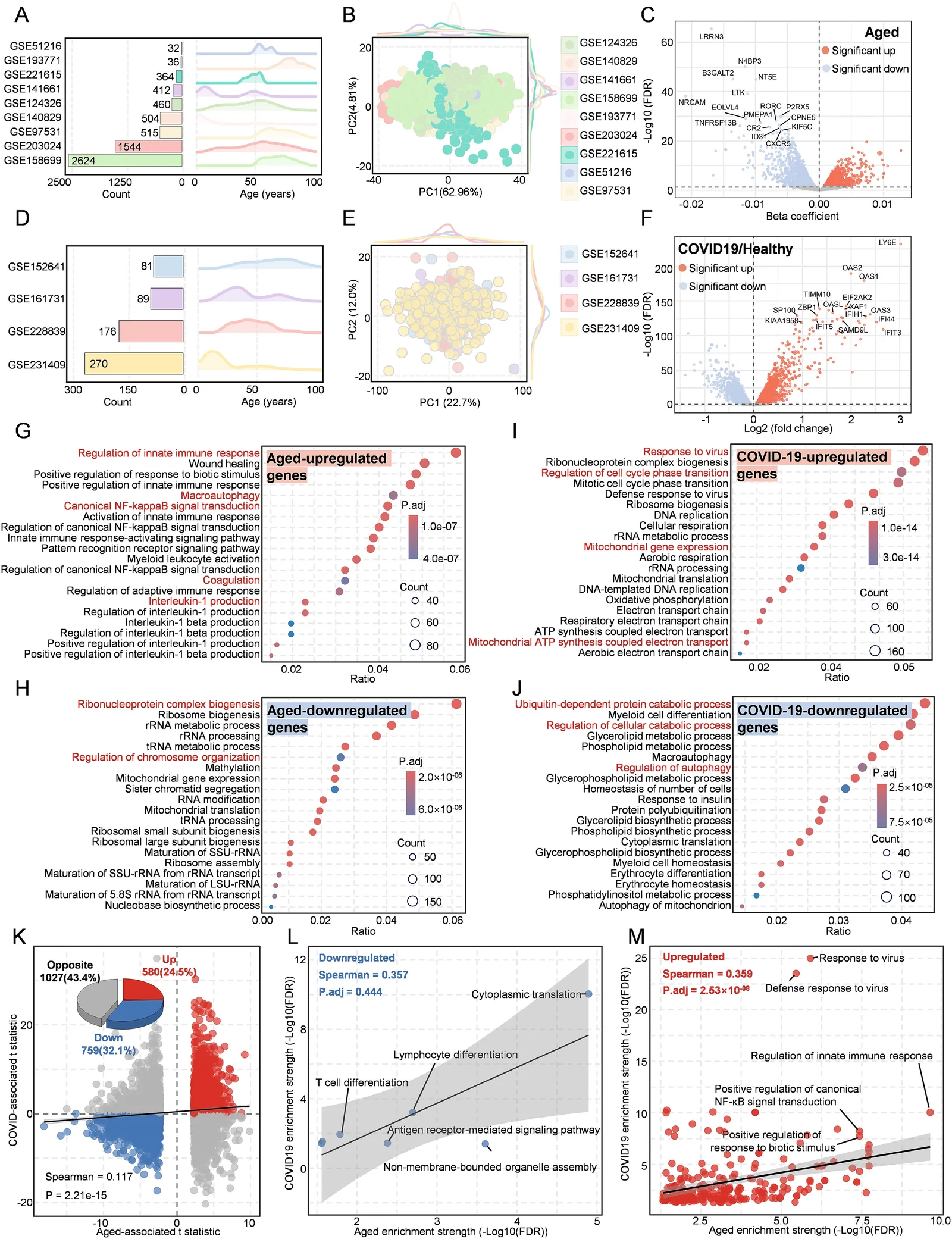
Integrative analysis of transcriptomic similarities and differences between aging and COVID-19. (A) Sample size and age distribution of nine healthy aging cohorts. Bar plots indicate sample numbers, and density plots show age distributions. (B) PCA of the integrated aging cohorts after batch-effect correction. (C) Volcano plot showing age-associated differential gene expression. (D) Sample size and age distribution of four COVID-19 cohorts. Bar plots indicate sample numbers, and density plots show age distributions. (E) PCA of the integrated COVID-19 cohorts after batch-effect correction. (F) Volcano plot showing COVID-19-associated differential gene expression. (G–J) GO enrichment analysis of significantly upregulated and downregulated genes in the aging and COVID-19 cohorts. (K) Gene-level similarity analysis between aging- and COVID-19-associated transcriptomic changes. (L–M) Similarity analysis of commonly upregulated and downregulated GO pathways between aging and COVID-19.

To further characterize COVID-19-associated transcriptomic alterations, we integrated four whole-blood COVID-19 transcriptomic cohorts to establish a COVID cohort comprising 469 patients with COVID-19 and 147 healthy controls (Fig. 1D). As performed for the Aging cohort, batch effects were corrected using neuroCombat while preserving age-related information (Fig. 1E). Subsequently, differential expression analysis was conducted using limma after adjusting for age, identifying 2748 downregulated genes and 3128 upregulated genes in COVID-19 patients relative to healthy controls (Fig. 1E-F, Supplementary Table S4). Notably, canonical interferon- and antiviral response-related genes, including *IFI44L*, *IFITM3*, *OAS1*, *OAS2*, and *ZBP1*, were markedly upregulated (Fig. 1F), indicating that the COVID cohort faithfully captures the characteristic peripheral blood immune transcriptomic responses induced by SARS-CoV-2 infection [22, 23].

Next, we performed Gene Ontology (GO) enrichment analysis on age-associated genes and COVID-19-associated differentially expressed genes to compare functional alterations between aging and COVID-19. Genes upregulated during aging were predominantly enriched in pathways related to innate immune response, type I interferon signaling, NF-κB signaling, and coagulation, whereas age-downregulated genes were mainly associated with RNA processing, ribonucleoprotein complex biogenesis, mitochondrial gene expression, and chromatin organization (Fig. 1G-H). Meanwhile, genes upregulated in COVID-19 were primarily enriched in response to virus and innate immune response pathways, while downregulated genes were mainly involved in protein catabolic processes and autophagy-related functions (Fig. 1I-J). Collectively, these findings indicate that COVID-19 and aging share partially overlapping transcriptomic functional signatures in peripheral blood, particularly characterized by enhanced innate immunity, interferon responses, and inflammatory processes [17, 22].

To systematically evaluate the relationship between COVID-19-associated transcriptional alterations and healthy aging, we further compared transcriptomic changes across all genes between the two conditions. We observed a modest yet highly significant positive correlation between COVID-19-associated effects and age-associated effects (Spearman’s *ρ* = 0.117, *P* = 2.21 × 10^−15^; Fig. 1K), suggesting an overall directional concordance between COVID-19-induced transcriptional changes and the aging process in peripheral blood (Supplementary Table S5). Based on this observation, we next examined the behavior of age-associated genes in COVID-19. A subset of aging-related genes exhibited concordant expression changes in COVID-19 patients. Specifically, genes including *BACH2*, *NELL2*, *PLXDC1*, and *TNFRSF13C*, which normally declined with age, were further downregulated in COVID-19, suggesting suppression of adaptive immune regulation, B-cell survival, and immune homeostasis-related programs. Conversely, genes such as *BATF2*, *KYNU*, *MARCO*, *STMN3*, and *TNFAIP6*, which increased with age, were also significantly upregulated in COVID-19, indicating coordinated activation of interferon responses, inflammatory signaling, and inflammation-associated tissue repair processes (Supplementary Fig. S1A-B) [22, 23].

At the pathway level, we further compared the trends of enriched pathways associated with COVID-19 and aging. The correlation among commonly downregulated pathways did not reach statistical significance (Spearman’s *ρ* = 0.357, *P* = 0.444; Fig. 1L), yet functions such as cytoplasmic translation and lymphocyte differentiation still showed a shared downward trend in both conditions. In contrast, commonly upregulated pathways exhibited stronger and statistically significant concordance (Spearman’s *ρ* = 0.359, *P* = 2.53 × 10^−8^; Fig. 1M), primarily involving response to virus, regulation of innate immune response, and NF-κB signaling, which are key processes related to innate immunity and inflammation [17, 22, 23].

These results suggest that COVID-19 may induce an aging-like molecular state in the peripheral blood immune system. However, differential expression and functional enrichment analyses can only describe the similarities between COVID-19 and aging, and cannot quantitatively assess the degree of aging-like changes at the individual level. Therefore, we further constructed an age-prediction model based on healthy peripheral blood transcriptomes to quantify molecular changes that deviate from the healthy aging trajectory under disease conditions.

### Construction of a BAP for quantifying aging-associated molecular states

Based on the transcriptomic similarities between COVID-19 and healthy aging, we further constructed the BAP to establish a peripheral blood molecular reference trajectory of healthy aging. The age predicted by BAP was defined as biological age (BioAge), the actual chronological age was defined as chronological age (ChronAge), and the deviation between BioAge and ChronAge was subsequently used to derive AgeAccel, thereby quantifying aging-like molecular deviations under disease conditions (Fig. 2A).

**Figure 2:**
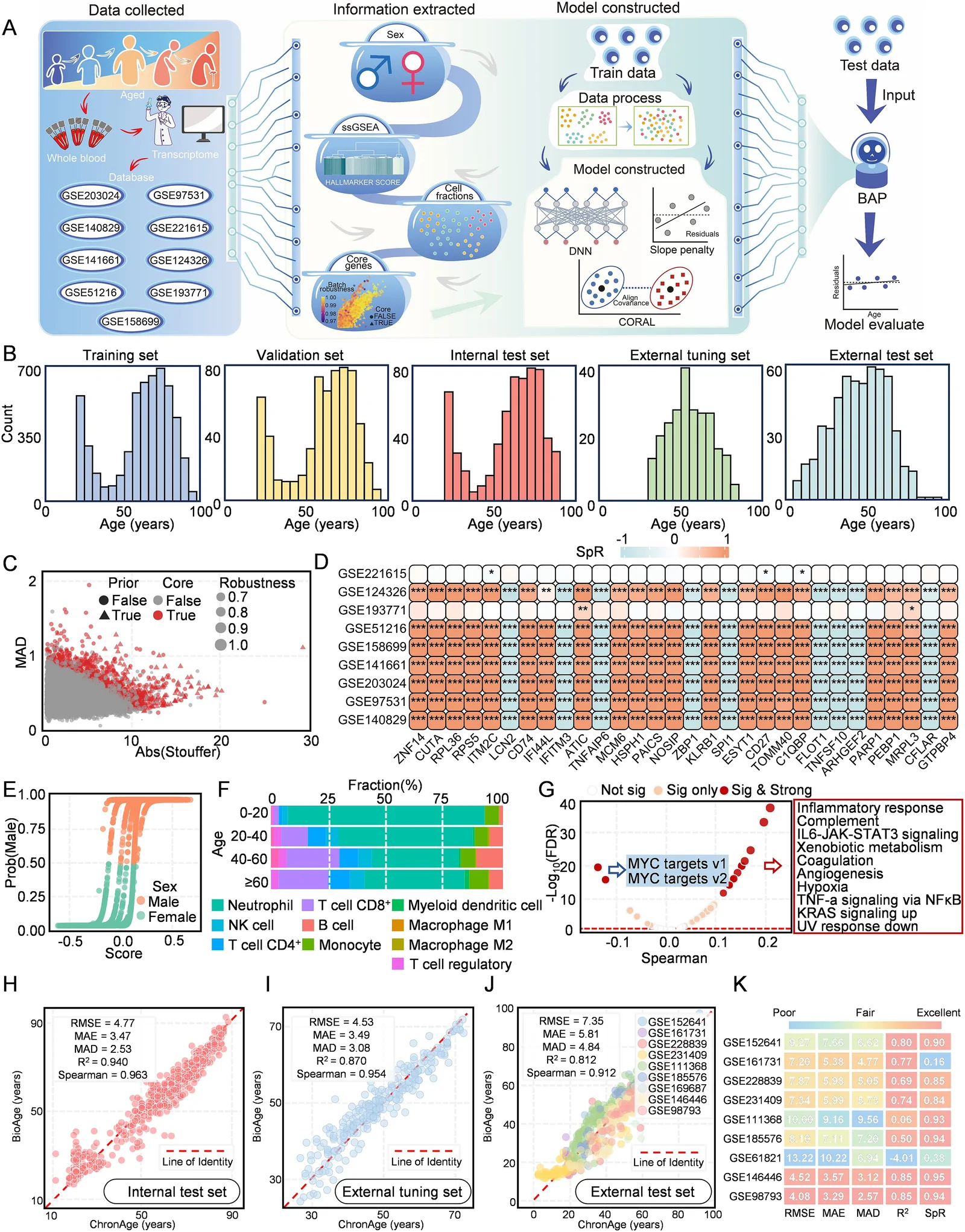
Construction and performance evaluation of the blood aging predictor (BAP). (A) Workflow of BAP model construction. (B) Sample size and age distribution of the training, validation, internal test, external tuning, and external test cohorts. Bar plots indicate sample numbers, and density plots show age distributions. (C) Scatter plot showing the selection of core age-associated genes. The x-axis represents the Stouffer score, the y-axis represents the MAD, and point size indicates cross-cohort robustness. (D) Heatmap showing Spearman’s correlations between gene expression and chronological age, together with statistical significance, for the top 30 genes across nine cohorts. (E) Sex prediction based on sex-associated marker genes. Probabilities >0.5 indicate male and probabilities <0.5 indicate female. (F) Age-associated changes in estimated peripheral blood cell composition. Bar plots show the trends of different cell types across age groups. (G) Volcano plot showing age-associated changes in ssGSEA pathway scores. Pathways with |Spearman| > 0.1 and − log10(FDR) > 10 were selected, resulting in 12 significantly age-associated pathways. (H–J) Predictive performance of BAP in the internal test set, external tuning cohort, and independent external test cohorts. Evaluation metrics are shown in the upper-left corner of each panel. (K) Heatmap summarizing BAP performance across nine independent external test cohorts.

Within the Aging cohort comprising 6491 samples, we first excluded samples with documented disease conditions, resulting in 5808 healthy individuals retained for model development. Given the substantial age-related batch effects across the multiple aging datasets and the differences in age distributions among datasets, we performed feature selection using samples from all aging datasets to provide a straightforward and interpretable framework for feature screening. These samples were subsequently divided into training, validation, and internal test sets at a ratio of 8:1:1. In parallel, we assembled an external test cohort comprising 530 healthy samples to evaluate the model’s generalizability across independent datasets. In addition, we established an independent healthy cohort consisting of 243 samples with a relatively balanced age distribution, which was used as an external tuning set to further optimize model performance and enhance generalizability (Fig. 2B; Supplementary Fig. S2A-D).

During feature selection, we first identified genes that were significantly associated with age and exhibited consistent age-related changes across multiple cohorts. By integrating age-association analysis with cross-cohort robustness assessment, we ultimately identified 398 high-confidence age-associated genes (Fig. 2C-D; Supplementary Table S6). These genes showed substantial overlap with previously reported blood aging markers identified by Peters et al. [17], further supporting the biological validity of this gene set (Supplementary Fig. S3A). To further determine whether these age-associated genes represented distinct expression patterns and functional modules, we performed Mfuzz clustering analysis on the 398 genes (Supplementary Fig. S3B-C; Supplementary Table S7) [24]. The analysis revealed three gene modules with distinct age-related expression patterns. Cluster 1, centered on *FBL*, *JAK2*, and *MYC*, was primarily enriched in ribosome biogenesis, rRNA metabolic processes, and protein kinase activity-related functions. Cluster 2, represented by *CD8A*, *PARP1* and *TNFRSF10B*, was mainly associated with phosphorylation binding, GTP regulation, and signal transduction-related functions. Cluster 3, centered on *KLF1*, *SPI1*, and *PTPRC*, was enriched in granule formation and lysosome-related processes, including primary lysosome, specific granule, and cytolytic granule pathways (Supplementary Fig. S3D-E).

In addition to gene expression features, we integrated multiple layers of biological information to construct a comprehensive predictive feature framework. First, sex information was inferred from the expression of sex-specific marker genes and incorporated into the model. Second, peripheral blood cell composition was estimated using transcriptomic deconvolution approaches [25]. In parallel, single-sample gene set enrichment analysis (ssGSEA) was employed to quantify the activity of age-associated functional pathways. Following feature selection, a final integrated feature set comprising 422 input variables was established, including 398 gene expression features, 11 cell composition features, 12 pathway activity features, and 1 sex feature (Fig. 2E-G; Supplementary Fig. S3F).

We systematically evaluated ten conventional machine-learning models together with four deep neural network (DNN) variants (Supplementary Fig. S4A). For the DNN model, we incorporated a slope penalty constraint and the correlation alignment (CORAL) algorithm to mitigate age-dependent prediction bias and domain shifts between the training data and the samples to be predicted. Model performance was assessed using multiple complementary metrics, including root mean square error (RMSE), mean absolute error (MAE), median absolute deviation (MAD), coefficient of determination (R^2^), and Spearman’s correlation, which collectively reflect prediction accuracy, robustness, goodness of fit, and concordance between predicted and chronological age. The results showed that the DNN model, optimized using CORAL and slope penalty strategies, consistently achieved the best performance across the internal test set, external tuning set, and independent external test datasets (Supplementary Fig. S4A). After determining the optimal hyperparameters, we performed model ensembling and parameter optimization based on the external tuning set, resulting in the final BAP (Supplementary Tables S8–10). The final model achieved a mean absolute error of 3.47 years and an R^2^ of 0.940 in the internal test set. Notably, because age-related features were selected using the combined samples from all aging datasets, the model performance observed in the internal test set may be overly optimistic and therefore not accurately reflect the true predictive performance of BAP. In the external tuning set, the model achieved an MAE of 3.49 years and an R^2^ of 0.870. Because the model was primarily optimized using the external tuning set, its performance on this dataset cannot accurately reflect the model’s generalizability. Across nine completely independent external cohorts, BAP achieved an MAE of 5.81 years and an R^2^ of 0.812 (Fig. 2H-K; Supplementary Fig. S4B-C; Supplementary Tables S11–13). We believe that the performance across multiple independent external datasets better reflects the model’s generalizability. Meanwhile, using the same training strategy as BAP except for the input features, we developed a gene-only model using only gene expression features. Across the external datasets, this model achieved an MAE of 6.58 years and an R^2^ of 0.809, showing slightly lower predictive performance than BAP, which additionally incorporated secondary derived features (Supplementary Fig. S4D-G). Furthermore, we applied the gene-weighted model developed by Peters et al. [17] to the same external datasets and found that BAP showed better predictive performance than the Peters model across all nine datasets (Supplementary Fig. S5A-B). Although performance varied modestly across external cohorts, BAP consistently demonstrated strong predictive accuracy and robust generalizability. These results indicate that BAP reliably captures the progressive transcriptomic alterations that accumulate during healthy aging in peripheral blood and provides a robust tool for quantifying disease-associated AgeAccel.

### BAP captures broad molecular features of aging-associated changes

To further elucidate the biological features learned by BAP during age prediction, we applied SHapley Additive exPlanations (SHAP) analysis to interpret the model predictions (Fig. 3A). SHAP analysis identified *SPTBN1*, *DOCK9*, and *RPL36* among the most important predictors of age, with *DOCK9* and *SPTBN1* exhibiting the strongest contributions to age prediction (Fig. 3B; Supplementary Table S14). These genes are associated with biological processes including cytoskeletal maintenance, cellular signal transduction, and ribosomal function [26, 27].

**Figure 3:**
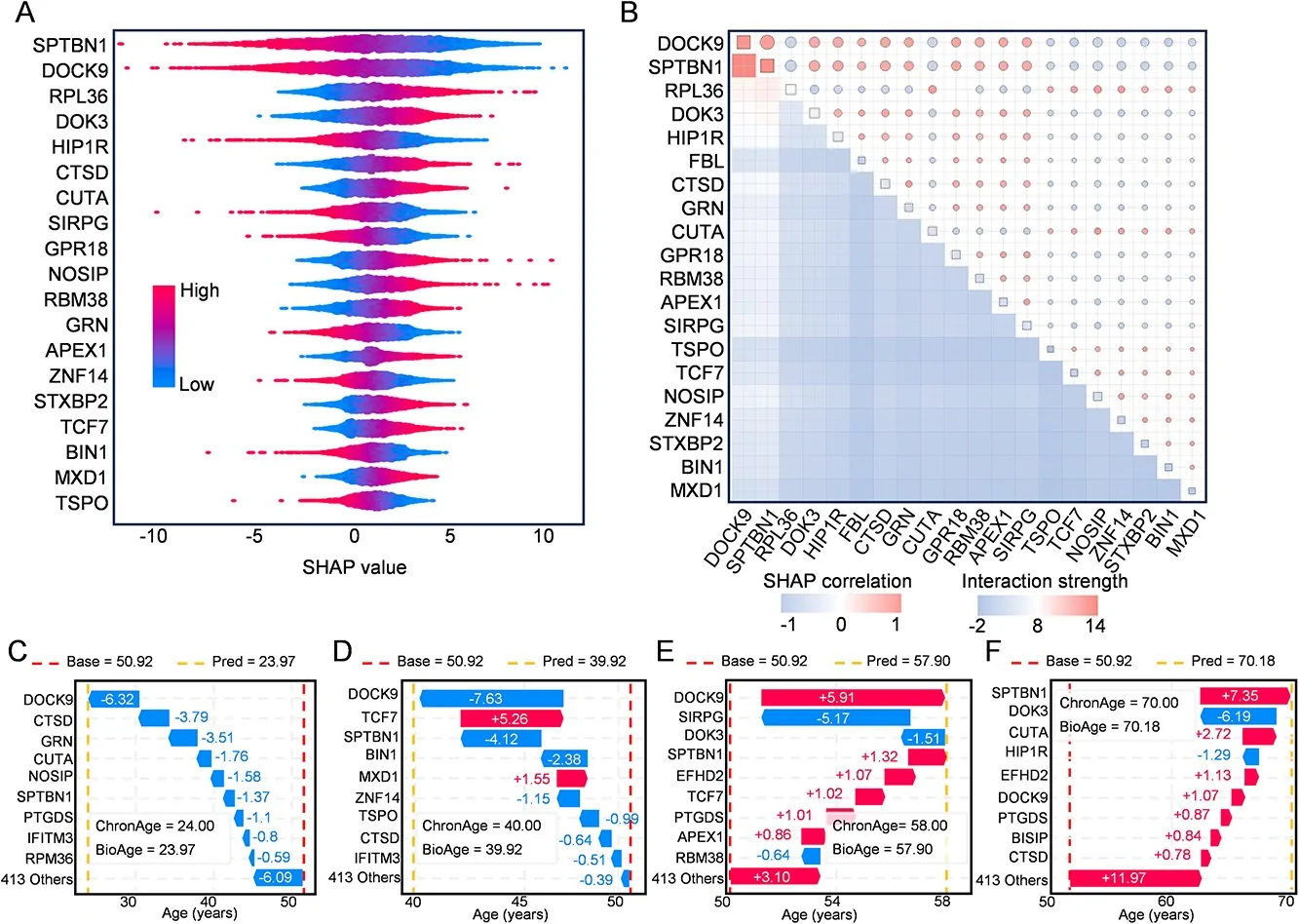
SHAP-based interpretation of the BAP model. (A) SHAP beeswarm plot showing the contributions of individual features to BAP predictions. (B) SHAP interaction heatmap showing interactions among key predictive genes and their contributions to model predictions. (C–F) SHAP contribution patterns of the top 10 predictive genes and the remaining features in representative individuals from four age groups.

To further understand how BAP generates age predictions across individuals of different ages, we examined SHAP contribution patterns at the individual level. The results showed that BAP does not determine predicted age based on a single feature. Instead, it first uses a small number of key features to establish an approximate age range and subsequently refines the prediction through the cumulative contributions of numerous auxiliary features (Fig. 3C-F). This prediction pattern suggests that aging-related signals in the peripheral blood transcriptome are not driven by a small set of marker genes alone but are jointly encoded by multiple molecular features. In other words, BAP integrates multidimensional information, including gene expression, cellular composition, pathway activity, and sex, thereby providing a more robust characterization of an individual’s aging-associated molecular state. Therefore, BAP is not merely a model for predicting chronological age but also provides a comprehensive framework for quantifying aging-associated molecular states in peripheral blood.

### COVID-19 induces transcriptomic AgeAccel across age groups

To evaluate the extent to which COVID-19 promotes aging-like molecular changes, we applied the final BAP model to the integrated independent COVID-19 transcriptome cohorts studied above (Fig. 4A; Supplementary Table S15). First, we decomposed the model predictions for COVID-19 samples using the SHAP method. The results showed that, in COVID-19 samples, predictions were not dominated by a few key features but rather relied on the collective contributions of many variables, suggesting that BAP primarily utilizes broad transcriptomic information to predict age rather than depending solely on inflammation- or immune-related signals (Fig. 4B).

**Figure 4:**
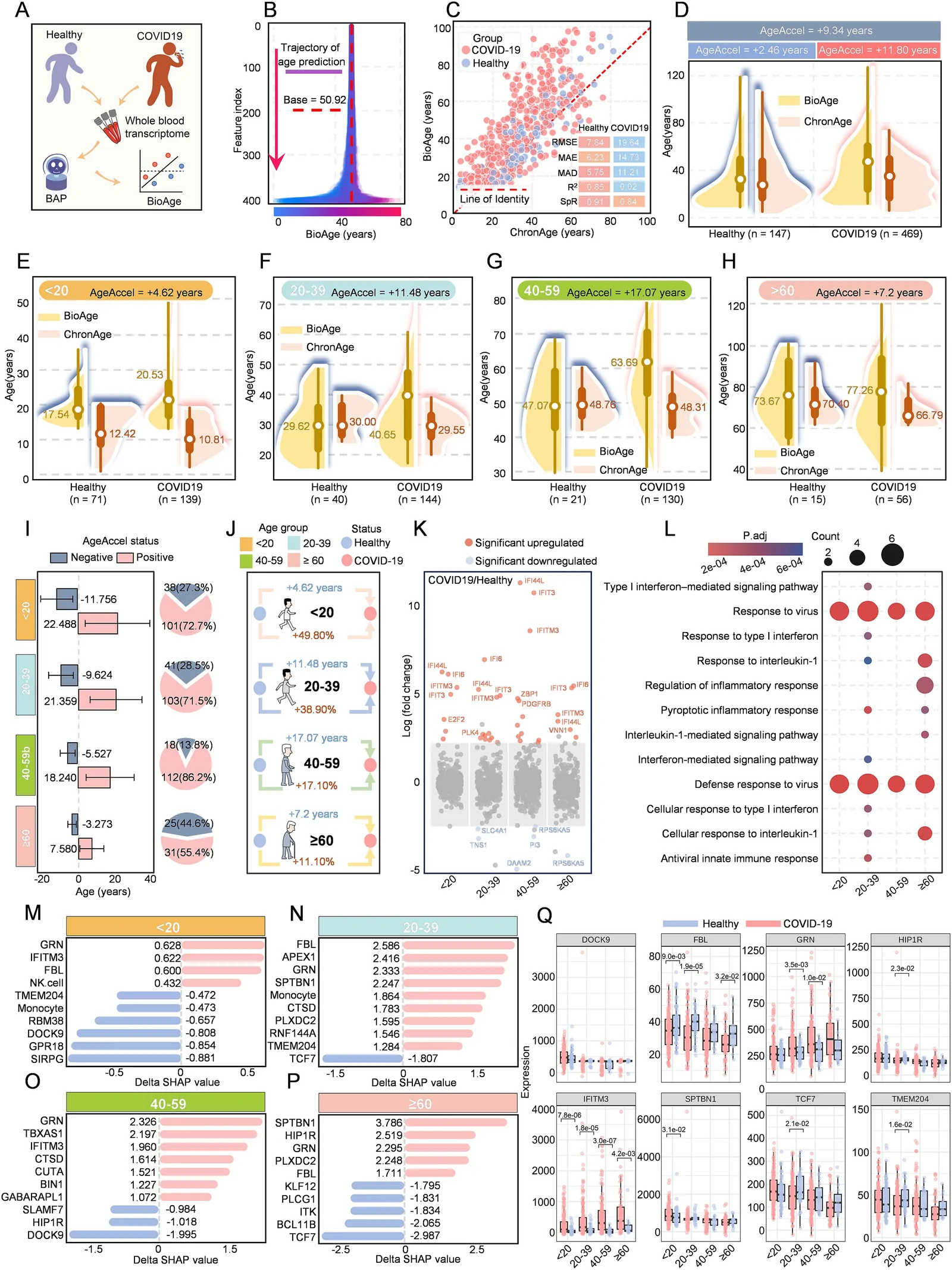
Evaluation of the impact of COVID-19 on BioAge. (A) Workflow for predicting BioAge in the COVID-19 cohort using the BAP model. (B) Prediction trajectory showing changes in model output as features are progressively incorporated during COVID-19 prediction. (C) Scatter plot comparing predicted BioAge between healthy controls and COVID-19 samples. The accompanying heatmap summarizes prediction performance in the two groups. (D) Violin plots showing age deviation in healthy controls and COVID-19 samples. (E–H) AgeAccel across different age groups stratified at 20-year intervals. Violin plots show age deviation in healthy controls and COVID-19 samples, and numbers indicate corrected AgeAccel values. (I) Summary of corrected age deviation across age groups. Bar plots show the direction and magnitude of age deviation, and pie charts show the proportions of samples with positive or negative age deviation. (J) Analysis of absolute and relative age deviation across age groups. The upper numbers indicate absolute age deviation, whereas the lower numbers indicate relative age deviation. (K) Volcano plots showing differentially expressed genes across four age groups. Genes were selected using |logFC| > 2 and FDR < 0.05. (L) GO enrichment analysis of differentially expressed genes across age groups. (M–P) SHAP contribution analysis of age prediction in COVID-19 samples across different age groups. (Q) Boxplots showing expression changes in eight representative key genes across different age groups. Group *P* values were calculated using the Wilcoxon rank-sum test and are indicated in the plots.

We next compared the distribution of predicted BioAge between healthy controls and COVID-19 patients. Compared with healthy controls, COVID-19 samples exhibited markedly elevated predicted ages, accompanied by substantially increased prediction errors (Fig. 4C). These findings suggest that the extensive molecular remodeling induced by COVID-19 may perturb the normal aging trajectory learned from healthy individuals, thereby shifting predicted age toward older values. After correcting for cohort-specific bias using the age deviation observed in healthy controls as a baseline, COVID-19 patients exhibited a mean AgeAccel of 9.34 years (Fig. 4D). Given that individuals of different ages may exhibit distinct molecular responses to COVID-19, we further stratified all samples into four age groups: <20 years, 20–39 years, 40–59 years, and ≥ 60 years. Significant AgeAccel was observed across all four age groups, indicating that COVID-19-induced aging-like molecular alterations are not restricted to a specific age population (Fig. 4E-H). However, the magnitude of AgeAccel varied substantially across age groups. Specifically, the 40–59-year group exhibited the largest absolute age deviation, whereas the <20-year group showed the highest relative AgeAccel (Fig. 4I-J), suggesting that COVID-19 induces transcriptomic AgeAccel across age groups and affects molecular aging trajectories.

To further dissect the molecular basis underlying BAP-predicted AgeAccel, we first examined differential expression of the 398 key genes used in the BAP model across COVID-19 samples from different age groups. Across all age groups, significant upregulation of interferon-related genes, including *IFI44L* and *IFITM3*, was observed, accompanied by downregulation of genes involved in signal transduction, cytoskeletal organization, and homeostatic regulatory processes, such as *RPS6KA5* and *DAAM2* (Fig. 4K). Functional enrichment analysis further revealed significant activation of pathways including response to virus and type I interferon signaling across all age groups (Fig. 4L).

We further applied SHAP analysis to identify the key features driving elevated predicted age in COVID-19 samples across different age groups (Supplementary Table S16). Although the most influential predictors varied among age strata (Fig. 4M-P), several genes, including *GRN*, *IFITM3*, *FBL*, *TMEM204*, and *HIP1R*, consistently emerged as major contributors across multiple age groups and displayed significant age-associated expression patterns during healthy aging (Fig. 4Q). Among them, *GRN* and *IFITM3* are primarily involved in interferon and inflammatory responses, whereas *FBL*, *TMEM204*, and *HIP1R* are associated with ribosome biogenesis, cellular homeostasis, and cytoskeletal/intracellular trafficking processes, respectively. Together with the GO enrichment results, these findings suggest that although COVID-19-associated AgeAccel is primarily recognized through antiviral and inflammatory programs, the final age prediction reflects coordinated alterations across multiple aging-related molecular features.

### AgeAccel reflects molecular severity in COVID-19

AgeAccel is generally considered to reflect the extent to which an individual’s biological state deviates from the normal aging trajectory [15, 28]. We therefore asked whether AgeAccel in COVID-19 patients carries disease-related biological significance beyond simply quantifying age deviation. To address this question, we stratified COVID-19 patients according to their degree of AgeAccel into a COVID fast-aging (CFA) group and a COVID slow-aging (CSA) group, and the detailed data-splitting strategy is described in the Methods section (Fig. 5A; Supplementary Fig. S6A-B). We then extended our analysis beyond the predictive features used by the BAP model to the whole-transcriptome level, aiming to determine whether CFA and CSA exhibit broader molecular differences across the transcriptome.

**Figure 5:**
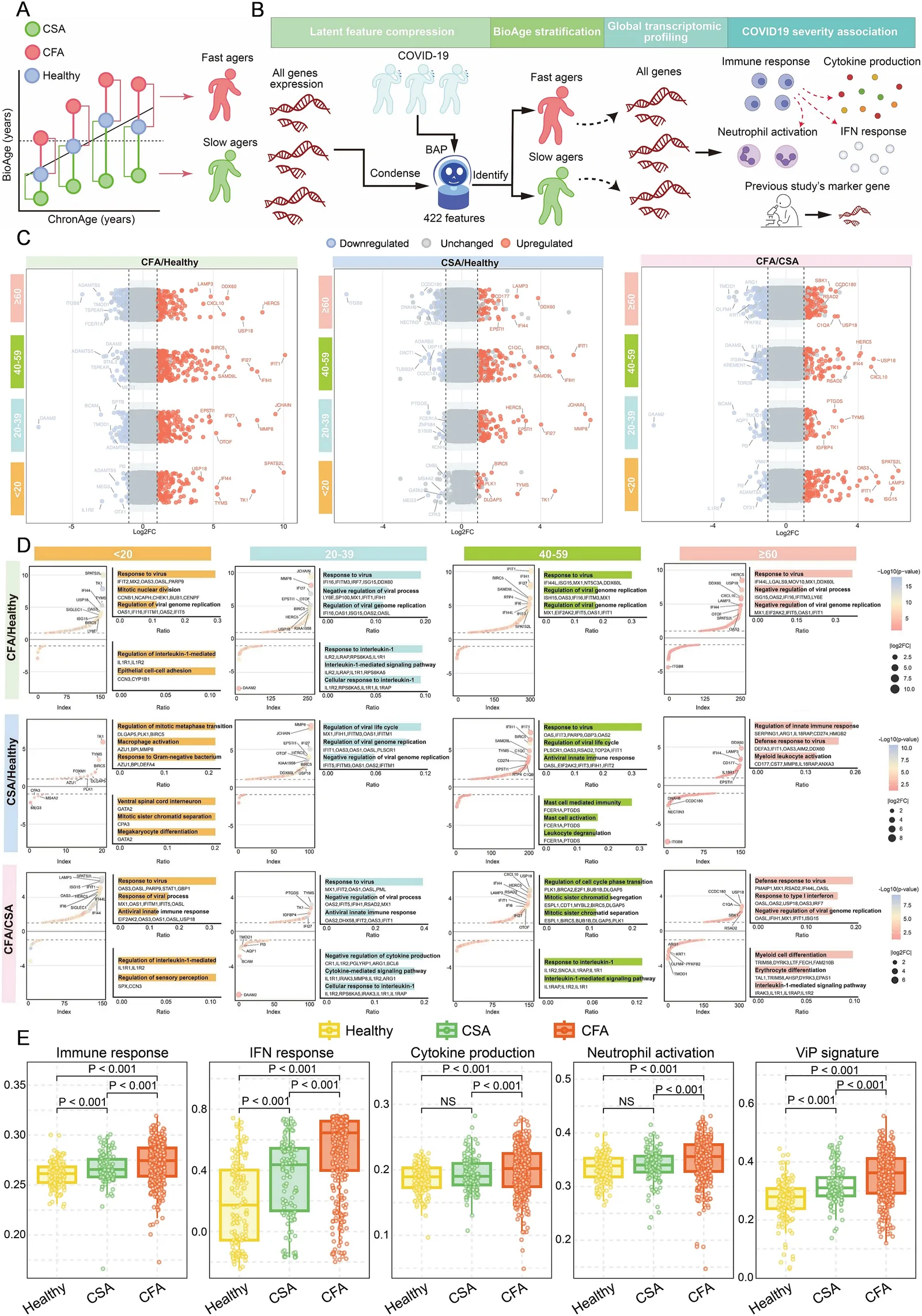
Subtype analysis of COVID-19-related AgeAccel. (A) Schematic illustration of the two COVID-19 AgeAccel subtypes, CFA and CSA. (B) Classification of CFA and CSA based on BAP-selected genes, followed by re-evaluation of transcriptome-wide molecular perturbations in COVID-19. (C) Volcano plots showing differentially expressed genes among CFA, CSA, and healthy controls across age groups. Genes were selected using the criteria |log2FC| > 1 and FDR < 0.05. (D) Summary of upregulated and downregulated genes across groups and age strata. (E) Comparison of enrichment scores for five COVID-19 severity- and inflammation-related gene sets. *P* values were calculated using the Wilcoxon rank-sum test and adjusted for multiple comparisons using the Benjamini–Hochberg method.

We first compared transcriptomic differences among CFA, CSA, and healthy samples. Both CFA and CSA exhibited typical COVID-19-associated molecular features compared with healthy controls, including upregulation of antiviral genes such as *IFIT1*, *IFI27*, and *EPSTI1*, and downregulation of homeostasis-related genes such as *ITGB8* and *DAAM2* (Fig. 5B-C; Supplementary Tables S17–S18). Direct comparison between CFA and CSA revealed that immune, inflammatory, and cell proliferation-related genes such as *IFI44*, *CXCL10*, and *TK1* remained elevated in CFA samples, suggesting that although both groups were affected by COVID-19, patients with higher AgeAccel exhibited stronger molecular perturbations (Fig. 5C; Supplementary Table S19). We next performed functional enrichment analysis of the differentially expressed genes. Compared with healthy controls, both CFA and CSA were significantly enriched for pathways related to response to virus, response to interferon, and response to interleukin-1. Notably, these pathways remained significantly different between CFA and CSA, accompanied by alterations in cytokine signaling and mitotic sister chromatid separation-related processes (Fig. 5D). These findings suggest that COVID-19 patients with higher AgeAccel exhibit more pronounced antiviral responses, inflammatory activation, and cell cycle-related molecular alterations.

To further evaluate whether AgeAccel reflects COVID-19 disease burden, we selected molecular severity features including immune activation, interferon response, cytokine production, neutrophil activation, and a previously reported viral pandemics (ViP) signature associated with COVID-19 severity, while confirming minimal overlap between these gene sets and the BAP key predictive features [29] (Fig. 5E; Supplementary Fig. S6C; Supplementary Table S20). The results showed that CFA samples exhibited significantly higher scores than CSA samples across all molecular severity modules, while CSA samples also exhibited significantly higher ViP scores than healthy controls (Fig. 5E; Supplementary Table S21). Furthermore, we treated AgeAccel as a continuous variable and evaluated its linear associations with different inflammation-related scores. Higher AgeAccel was associated with higher inflammatory ssGSEA scores and higher ViP scores (Supplementary Fig. S6D). These findings indicate that AgeAccel consistently correlates with COVID-19-related molecular severity.

Notably, the differences between CFA and CSA, as well as the changes associated with increased AgeAccel, were not limited to antiviral and inflammatory responses. Further analysis of canonical aging-related functional modules revealed that, compared to CSA, CFA samples exhibited elevated DNA damage response and unfolded protein response (UPR), accompanied by decreased macroautophagy and mitophagy activity (Supplementary Figs S6E and S7A-B; Supplementary Tables S22–23). These changes are highly consistent with hallmarks of aging, including impaired genomic stability, increased proteostasis stress, and reduced cellular quality control [4, 30, 31]. However, no significant differences in cellular senescence scores were observed between CFA and CSA, suggesting that COVID-19-associated AgeAccel does not fully correspond to classical accumulation of cellular senescence, but rather reflects infection-induced systemic molecular homeostasis imbalance. In summary, AgeAccel is closely associated not only with COVID-19-related immune and inflammatory responses, but also with dysregulation of multiple canonical aging programs, indicating its potential as a molecular metric of COVID-19-related molecular burden and disease severity.

### COVID-19 induces a potentially reversible aging-like molecular state at the cohort level

To further investigate the dynamic relationship between disease progression and AgeAccel, we analyzed a longitudinal transcriptomic dataset including samples collected before infection (Pre, *n* = 58), during acute infection (First, *n* = 49), at an intermediate recovery stage (Mid, *n* = 351), and after recovery (Post, *n* = 430) (Supplementary Fig. S8A-D) [32]. Because complete subject-level identifiers allowing reliable matching of samples across different stages were unavailable, we evaluated AgeAccel across disease stages at the cohort level. First, we applied the BAP model to predict age across different stages. The results showed that AgeAccel increased significantly during the acute infection phase (First), then gradually declined during recovery, and approached pre-infection levels in the Post phase. Compared to the Pre stage, only ~1.25 years of AgeAccel remained in the Post stage, suggesting that COVID-19-induced AgeAccel is not permanent but may be partially reversible following recovery (Supplementary Fig. S8A-F; Supplementary Table S24). This estimate reflects the average difference between the Post and Pre groups rather than a within-subject residual effect, indicating a substantial reduction in AgeAccel after recovery compared with the acute infection stage.

We further assessed the disease severity-related molecular features described above to explore the biological basis underlying this dynamic change (Supplementary Fig. S8G-H). The results showed that these functional modules were markedly elevated during the acute infection phase and gradually returned to near-baseline levels during recovery, indicating that the dynamic changes in AgeAccel align with the fluctuations in COVID-19-related immune and inflammatory burden (Supplementary Fig. S8G).

We next analyzed the aging-related functional modules described above across different disease stages. The results showed that cellular senescence scores were significantly elevated during the First and Mid stages compared with the Pre stage, but returned to baseline levels in the Post stage (Supplementary Fig. S8H; Supplementary Table S25). Meanwhile, functional modules including DNA damage response, macroautophagy, mitophagy, TNF-α/NF-κB signaling, and the UPR also exhibited marked dynamic changes and progressively returned toward normal levels during recovery (Supplementary Fig. S8H; Supplementary Table S26). These findings indicate that COVID-19 infection is accompanied not only by increased predicted biological age but also by coordinated perturbations across multiple aging-associated molecular programs, with AgeAccel both capturing aging-like molecular changes and disease severity in a stage-dependent manner.

### AgeAccel captures disease-associated molecular burden across diseases

In COVID-19, AgeAccel not only reflects aging-like molecular alterations but is also closely associated with disease severity and recovery dynamics. To determine whether AgeAccel merely represents a COVID-19-specific inflammatory response or captures broader disruptions of molecular homeostasis across disease states, we extended our analysis to cohorts of sepsis, influenza, and major depressive disorder (MDD) (Supplementary Fig. S9A-F; Supplementary Tables S27–29). This analysis enabled us to evaluate whether AgeAccel can serve as an aging-referenced molecular homeostasis metric for quantifying deviations from healthy physiological states across diverse disease contexts.

We first applied the BAP model to sepsis and influenza cohorts. Both infectious diseases exhibited significant AgeAccel (Supplementary Fig. S9A-D). To explore the relationship between AgeAccel and molecular features, we stratified individuals into fast- and slow-aging groups: sepsis fast-aging versus sepsis slow-aging (SSA) and influenza fast-aging versus influenza slow-aging. Individuals in the fast-aging groups showed enhanced interferon (IFN) response, cytokine production, and immune activation compared with their corresponding slow-aging groups, consistent with observations in COVID-19 and indicating that AgeAccel is closely linked to elevated immune-inflammatory burden and systemic molecular perturbation across infectious diseases (Supplementary Fig. S9G-J; Supplementary Tables S30–31).

To further characterize aging-related molecular features captured by AgeAccel, we compared canonical aging-associated functional modules across AgeAccel groups after adjusting for chronological age. Fast-aging individuals exhibited significantly elevated DNA damage response and UPR, accompanied by reduced macroautophagy and mitophagy activity, compared with their slow-aging counterparts (Supplementary Fig. S9G-J; Supplementary Tables S32–33). Cellular senescence scores were also significantly higher in fast-aging groups. These molecular alterations closely mirrored those observed in the CFA group, indicating that despite distinct etiologies and pathological processes, high-AgeAccel states across infectious diseases converge on common aging-related abnormalities, including impaired genomic stability, increased proteostatic stress, compromised cellular quality control, and enhanced cellular senescence signaling.

We next analyzed the non-infectious disease cohort of major depressive disorder (MDD) (Supplementary Fig. S9E-F). Unlike sepsis and influenza, MDD did not exhibit significant AgeAccel, nor did it show markedly elevated IFN response, immune activation, or neutrophil activation signatures (Supplementary Fig. S9K-L; Supplementary Table S34). However, compared with the MDD slow-aging group, individuals in the MDD fast-aging group exhibited significantly elevated cellular senescence and DNA damage response scores (Supplementary Fig. S9K-L; Supplementary Table S35). Notably, the macroautophagy and mitophagy abnormalities observed in infectious diseases were absent in the MDD cohort, suggesting that the specific aging-related molecular modules associated with AgeAccel vary across disease contexts. More importantly, these findings further demonstrate that AgeAccel is not merely a surrogate marker of infection-associated inflammation or interferon activation. Even in a non-infectious disease lacking pronounced immune-inflammatory activation, elevated AgeAccel was still associated with molecular abnormalities related to cellular senescence and DNA damage responses. Therefore, we propose that AgeAccel may represent the extent to which multiple aging-related molecular programs collectively deviate from normal homeostasis during disease. AgeAccel can serve as a composite molecular metric anchored to aging, enabling the quantification of disease-associated systemic molecular burden, deviations in overall health status, and disruptions in molecular homeostasis.

## Discussion

This study establishes healthy blood transcriptomic aging as a reference trajectory for quantifying disease-induced molecular deviation. Aging is increasingly understood not simply as a chronological process, but as a progressive loss of molecular homeostasis across interconnected biological systems [2, 4, 5, 33]. Among these systems, the immune compartment is particularly relevant because age-related immune remodeling, including impaired adaptive immunity, aberrant innate immune activation, chronic inflammation, and altered immune-cell composition, directly shapes disease susceptibility and recovery capacity [4, 34]. Peripheral blood therefore provides an accessible and informative window into systemic immune and inflammatory states [18, 35, 36]. By constructing a BAP from healthy individuals, our study reframes transcriptomic AgeAccel from a conventional residual of age prediction into an interpretable measure of how far an individual’s molecular state deviates from the expected trajectory of healthy aging.

The dynamic nature of the blood transcriptome is central to this framework. Biological age clocks based on DNA methylation, proteomics, metabolomics, transcriptomics, and multi-omics features capture distinct layers of aging biology [14, 16, 18, 19, 28]. DNA methylation clocks are highly stable and often reflect long-term epigenetic remodeling, whereas proteomic and metabolomic clocks may capture systemic inflammatory, secretory, and metabolic alterations [14, 16, 28]. In contrast, transcriptomic clocks are sensitive to rapidly changing immune-cell states, gene-expression programs, and pathway activities, making them particularly suitable for assessing acute infection, inflammation, and disease-associated molecular perturbations [18]. Thus, transcriptome-derived AgeAccel should not be interpreted as irreversible biological aging. Rather, it represents an aging-referenced molecular deviation that reflects the extent to which peripheral immune and transcriptional homeostasis departs from healthy age-expected patterns.

In COVID-19, SARS-CoV-2 infection induced marked transcriptomic AgeAccel across age groups and was associated with interferon signaling, immune activation, cytokine-related programs, neutrophil-associated responses, and molecular features of disease burden. These findings suggest that COVID-19 does not simply activate isolated antiviral pathways, but induces a broader remodeling of blood molecular homeostasis that partially converges with aging-associated programs. Importantly, longitudinal analysis showed that AgeAccel increased during acute infection and declined during recovery, suggesting that COVID-19-associated aging-like molecular changes may be partially reversible rather than representing a permanent acceleration of chronological aging. However, the longitudinal dataset lacked complete subject information to reliably match samples from the same individuals across different time points. Therefore, this analysis reflects group-level changes rather than within-subject residual effects of COVID-19. This distinction is essential: the 9.34-year average AgeAccel observed in COVID-19 should be viewed as an apparent transcriptomic displacement along an aging-referenced coordinate, not as evidence that infection permanently advances organismal aging by the same amount. Future cohorts with detailed clinical annotations and long-term follow-up will be needed to determine whether persistent AgeAccel after recovery predicts post-acute sequelae, incomplete immune restoration, or long-term health risks.

The extension of this framework to sepsis, influenza, and major depressive disorder further supports the broader utility of AgeAccel as a disease-associated molecular metric. In infectious diseases, elevated AgeAccel was accompanied by enhanced immune-inflammatory activation and aging-related stress programs, suggesting that systemic infection can drive shared aging-referenced molecular states despite distinct etiologies. In contrast, major depressive disorder did not show significant cohort-level AgeAccel, yet the high-AgeAccel subgroup displayed senescence-associated and genomic instability-related signals without strong interferon or immune-activation signatures. This observation argues against viewing AgeAccel as merely a surrogate of antiviral or inflammatory responses. Instead, AgeAccel appears to integrate context-dependent alterations across multiple aging-related modules, including immune activation, cellular stress, senescence, genomic instability, and impaired homeostatic regulation. Overall, our study proposes that healthy aging trajectories can serve as a biological coordinate system for quantifying disease-induced disruption of molecular homeostasis. With further validation in prospective, clinically annotated, and multi-omics cohorts, blood transcriptomic AgeAccel may provide a practical framework for assessing systemic disease burden, monitoring recovery dynamics, and identifying individuals whose molecular health state is disproportionately shifted away from healthy age-expected patterns.

An important limitation is that the current BAP framework relies on cohort-level normalization and batch correction and was therefore not designed for direct application to a single new sample in the absence of appropriate reference data. Future development of single-sample-compatible normalization and calibration strategies will be necessary for prospective clinical application.

## Materials and methods

### Data collection and dataset partitioning

All data collection, preprocessing, and dataset partitioning procedures were performed in R (v4.3.2). Whole-blood transcriptomic datasets were obtained from the Gene Expression Omnibus (GEO) database. In total, 23 publicly available cohorts were collected, including GSE203024, GSE97531, GSE140829, GSE221615, GSE141661, GSE124326, GSE51216, GSE193771, GSE158699, GSE177044, GSE111368, GSE61821, GSE185576, GSE146446, GSE98793, GSE152641, GSE161731, GSE228839, GSE231409, GSE185263, GSE66175, GSE169687, and GSE198449.

Among these, nine datasets (GSE203024, GSE97531, GSE140829, GSE221615, GSE141661, GSE124326, GSE51216, GSE193771, and GSE158699) were designated as internal cohorts for model development. After excluding samples with documented disease conditions, healthy samples were balanced across batches and age strata and randomly divided into training, validation, and internal test sets at a ratio of 8:1:1.

GSE177044 was used as an external tuning cohort for model selection and hyperparameter optimization. Independent external test was performed using nine datasets, including GSE111368, GSE61821, GSE185576, GSE146446, GSE98793, GSE152641, GSE161731, GSE228839, and GSE231409.

In addition, four datasets (GSE185263, GSE66175, GSE169687, and GSE198449) were reserved as independent external cohorts for downstream prediction and exploratory analyses.

### Data preprocessing

The 23 transcriptomic datasets comprised 10 microarray cohorts and 13 RNA-seq cohorts. Microarray datasets were annotated using platform-specific annotation files, whereas RNA-seq datasets were uniformly re-annotated using Ensembl BioMart (v2.58.2) to ensure consistent gene identifiers across datasets [37]. To minimize platform-specific differences in gene detection and avoid bias due to missing features, only genes with nonzero expression in more than 90% of samples across all datasets were retained, resulting in a total of 11 366 genes for downstream analyses.

For RNA-seq datasets, raw count data were first converted to transcripts per million, followed by log2 transformation. To harmonize distribution differences between RNA-seq and microarray datasets, quantile normalization was performed separately within each cohort using preprocessCore (v1.64.0) [38]. Batch effects were corrected using neuroCombat (v1.0.14), with age included as a covariate to preserve age-related biological signals [20]. Specifically, quantile normalization was first performed separately for each of the cohorts used. The internal and external datasets were then integrated separately and corrected using neuroCombat with age as a covariate. All processed datasets were subsequently integrated into a unified expression matrix for downstream feature extraction and model development.

### s‌sGSEA analysis

ssGSEA was performed using gene sets from the molecular signatures database (MSigDB) Hallmark collection (v7.5.1), retaining pathways containing 10 to 500 genes [39]. Enrichment scores were calculated using Gene Set Variation Analysis (GSVA) (v1.48.3) [40]. To minimize technical biases arising from multi-batch data, ssGSEA scores were computed independently within each batch. Subsequently, the resulting pathway scores were centered and scaled within each batch.

### Construction of sex covariate

The sex covariate was inferred based on canonical sex-related marker genes in the expression profiles. Y chromosome-associated genes, including *RPS4Y1*, *KDM5D*, *DDX3Y*, and *EIF1AY*, as well as X chromosome inactivation-related genes, including *XIST*, *TSIX* and *DXZ4*, were extracted, and their expression values were rank-normalized within each batch. Subsequently, a sex score for each sample was defined as the difference between the mean expression of Y chromosome-associated genes and the mean expression of X chromosome inactivation-related genes. Finally, a two-component Gaussian mixture model was used to estimate the probability of each sample being male.

### Selection of age-related core genes

Age-related core genes were identified from the internal datasets. For each gene, Spearman correlations between gene expression and chronological age were calculated within each batch, and batch-level correlations were integrated using a weighted Stouffer’s method to obtain an overall association score. In addition, a composite effect size and a measure of robust variability, quantified by the median absolute deviation, were computed. Cross-batch robustness was assessed using a Kruskal–Wallis test on global rank regression residuals to evaluate batch effects, which were subsequently converted into a stability metric. The absolute association strength, variability, and batch stability were normalized and combined with equal weighting into a composite score. Genes were ranked according to this composite score, and highly correlated genes (Spearman correlation ≥0.85) were removed to reduce redundancy. This procedure ultimately yielded 398 core genes for downstream analyses.

### Mfuzz clustering

Samples were first grouped by chronological age in 10-year intervals, and the mean expression within each age group was calculated to generate a gene × age-group expression matrix. Mfuzz analysis was then performed using Mfuzz (v2.60.0) [24]. The expression matrix was converted into an ExpressionSet object, followed by missing value filtering with a threshold of 0.25, mean imputation, and expression standardization. The fuzzification parameter (m) was estimated using the mestimate function. The optimal number of clusters was determined as three based on the elbow method, and a fuzzy c-means clustering with three clusters was applied to identify gene modules exhibiting similar age-related expression patterns.

### Machine learning and deep learning environment

All machine learning and DNN models were implemented in Python (v3.9.19). Conventional machine learning models, including linear models, tree-based models, and ensemble learning approaches, were developed using scikit-learn (v1.6.1). Ensemble algorithms were implemented with their respective Python libraries, including XGBoost (v2.1.4), LightGBM (v4.6.0), and CatBoost (v1.2.8). DNN models were built using TensorFlow (v2.20.0) and designed and optimized through the Keras high-level API. Data processing and analysis were primarily conducted using NumPy (v1.26.4) and pandas (v2.2.2).

### Machine learning model construction

A total of 10 regression models were implemented, including linear regression, Ridge regression, Lasso regression, ElasticNet regression, decision tree regression, random forest regression, support vector regression, XGBoost, LightGBM, and CatBoost. All models were trained and evaluated using 10-fold cross-validation with a fixed random seed of 42 to ensure reproducibility. For linear models, linear regression was applied with default parameters. Ridge regression was implemented with a regularization parameter *α* = 1.0. Lasso regression was configured with *α* = 0.01 and a maximum of 20 000 iterations, while ElasticNet was applied with *α* = 0.01, l1_ratio = 0.5, and a maximum of 20 000 iterations. For tree-based models, decision tree regression was used with default settings, and random forest regression was implemented with 300 trees. Support vector regression was performed using a radial basis function kernel, with parameters set to C = 10 and gamma = scale. For ensemble learning models, XGBoost was configured with n_estimators = 500, learning_rate = 0.03, max_depth = 6, subsample = 0.8, and colsample_bytree = 0.8. LightGBM was implemented with n_estimators = 500, learning_rate = 0.03, num_leaves = 31, subsample = 0.8, and colsample_bytree = 0.8. CatBoost was configured with 500 iterations, depth = 6, learning_rate = 0.03, and RMSE as the loss function.

### Deep learning model construction

Four DNN-based models were constructed, including a baseline model, a CORAL-regularized model, a slope penalty-regularized model, and a combined model incorporating both constraints. All models shared the same network architecture and training strategy, differing only in the loss function. Hyperparameters were optimized using the baseline model and applied consistently across all variants. The final model was a fully connected feedforward network with two hidden layers of 256 and 128 neurons using rectified linear unit activation, with dropout (0.35) and L2 regularization (1 × 10^−5^). The model was trained using the Adam optimizer (learning rate 0.0012, batch size 32) for up to 200 epochs with early stopping (patience = 25) under a 10-fold cross-validation framework. The baseline model optimized mean squared error, while the CORAL-regularized model introduced a CORAL loss with a weight of 0.01, and the slope penalty-regularized model imposed a slope constraint with a weight of 0.01. The combined model integrated both constraints.

### BAP model ensemble

The BAP model was constructed based on a DNN with CORAL and slope penalty constraints. Specifically, 10-fold cross-validation was performed on the training set and repeated 10 times, resulting in a total of 100 DNN models. Model performance was evaluated using the coefficient of determination and Spearman correlation on the validation folds, and models were ranked according to their cross-validation performance. The top 15 models were selected as candidates for ensemble construction. Model predictions were subsequently combined on the external tuning dataset using a constrained optimization approach to minimize the RMSE, with weights estimated via sequential least squares programming. The final ensemble prediction was obtained as a weighted average of the selected models and evaluated on the external test set.

### SHAP-based model interpretation

All analyses were performed in Python (v3.9.19) using the SHAP (v0.49.1) package [41]. The original feature matrix from the training set was used as input, with the corresponding labels as the response variable. For each DNN submodel in the ensemble, the trained model parameters and associated preprocessors were loaded, and input features were transformed accordingly to ensure consistency. The model was then redefined within the TensorFlow framework to standardize input–output mapping, and SHAP values were computed using the GradientExplainer. For each submodel, feature importance was quantified by averaging the absolute SHAP values across all samples, defined as the mean absolute SHAP value. At the ensemble level, feature importance scores from all submodels were aggregated and averaged by feature to obtain a global importance ranking for the entire model.

### Definition of disease-associated AgeAccel

AgeAccel was defined as the difference between predicted biological age, estimated by the BAP model, and chronological age. To account for systematic bias across datasets, the mean AgeAccel of healthy control samples within each dataset was used as a baseline offset. Disease-associated AgeAccel was then obtained by subtracting the corresponding dataset-specific baseline from the AgeAccel of disease samples.

### GO enrichment analysis

GO enrichment analysis was performed using the R package clusterProfiler (v4.14.6) [42]. Input gene symbols were deduplicated and converted to Entrez IDs using the org.Hs.eg.db (v3.20.0) database. Only gene sets containing at least 10 genes were analyzed. The Biological Process category was considered, and overrepresentation analysis was conducted using the enrichGO function [43]. *P*-values were adjusted for multiple testing using the Benjamini–Hochberg method, and entries with adjusted *P* < 0.05 were considered statistically significant.

### Correlation analysis between genes and pathways

Gene-level analysis: Age-related analysis results and COVID-19 differential expression results (adjusted for age) were first merged by gene name, and the corresponding statistics for each gene were extracted from both analyses. Age-significant genes were defined as those with FDR < 0.05. Spearman correlation was then used to assess the concordance between the age-related t-statistics and the COVID-19-related t-statistics. Based on the direction of the t-statistics in both conditions, genes were classified as concordantly upregulated, concordantly downregulated, or discordant. Genes that satisfied FDR < 0.05 in both age and COVID-19 analyses and exhibited consistent t-statistic directions were defined as co-varying genes.

GO-level analysis: Functional analysis was performed using the GO enrichment results of the respective gene sets. For each GO term, enrichment strength was defined as -log10(FDR), where FDR refers to the Benjamini–Hochberg adjusted *P*-value. Shared GO terms between two gene sets were identified based on their GO IDs, and the enrichment strengths in both sets were extracted. Spearman correlation was then used to evaluate the concordance of enrichment strengths across shared functional terms. The similarity between the two conditions at the functional level was quantified using the Spearman correlation coefficient, *P*-value, and the number of shared GO terms.

### Immune cell deconvolution analysis

Immune cell composition was estimated using the quanTIseq method implemented in the immunedeconv (v2.1.0) R package [25]. The batch-corrected expression matrix was first matched with sample metadata and converted into a gene × sample format. Genes with zero expression across all samples were removed, and the resulting expression matrix was input into quanTIseq to infer immune cell proportions. The estimated cell fractions were then organized by sample and matched with the corresponding batch and group information.

### Differential gene expression analysis

Age-related gene analysis was performed using the limma (v3.62.2) R package [21]. For healthy samples, chronological age was included as a continuous variable in the linear model with the formula: expression ∼ age.

For each gene, a linear model was fitted, and empirical Bayes moderation was applied to shrink the variance estimates. Age-related effects for each gene were quantified by regression coefficients, t-statistics, *P*-values, and Benjamini–Hochberg adjusted FDRs.

Disease-related differential expression analysis was also conducted using limma. Healthy controls were used as the reference group, and disease status was modeled as the main variable while including age as a covariate, with the formula: expression ∼ disease status + age.

Regression coefficients corresponding to disease status were extracted to represent expression changes relative to healthy controls. Differential expression significance was assessed using empirical Bayes-moderated statistics and adjusted for multiple testing using the Benjamini–Hochberg method.

### Definition of FA and SA groups

FA and SA groups were defined after correction for age-dependent model prediction bias. Healthy samples from each disease dataset were first used to fit an age-dependent prediction-bias curve using the smooth.spline function in the R stats package. The fitted curve was then used to correct the expected prediction bias of each disease sample according to its chronological age. Samples with a positive corrected prediction deviation were classified as FA, whereas those with a negative corrected deviation were classified as SA. This correction procedure was applied exclusively to FA/SA classification.

### Construction of immune, aging, and COVID-19 severity-related gene sets

Gene sets related to aging, inflammation, and COVID-19 severity were primarily curated from the MSigDB and published literature, and human gene sets were retrieved using the msigdbr (v25.1.1) R package [39]. Inflammation- and immune-related gene sets included IFN response, neutrophil activation, cytokine production, and immune activation. Among these, the IFN response gene set was manually defined based on canonical interferon-stimulated genes, including *IFIT1*, *IFIT3*, *ISG15*, *MX1*, *OAS1*, *OAS3*, *IFI27*, *STAT1*, *IFI6*, and *IFI44L*.

The COVID-19 severity-related feature set was defined using a previously reported ViP signature, including *CLIC4, CARD16, CARD17, HIST2H2AA4, HIST1H2AJ, SQRDL, HIST1H2AD, C21orf91, B2M, HIST2H2AB, HRASLS2, GCA, CCNA1, CASP1, GCH1, TRIM25, TMEM92, IFI27, LOX,* and *ELOVL7* [29].

Aging-related functional gene sets included cellular senescence, DNA damage response, macroautophagy, mitophagy, oxidative phosphorylation, reactive oxygen species response, unfolded protein response, and TNF-α/NF-κB signaling, which were extracted from the GO Biological Process or Hallmark collections of MSigDB. All gene sets were restricted to *Homo sapiens*, and duplicated genes were removed after extraction.

**Key Points**

- Developed a blood transcriptomic aging clock.
- Defined AgeAccel to measure disease-related aging deviation.
- Found increased AgeAccel in COVID-19 and other infections.
- Showed COVID-19-induced AgeAccel can decline during recovery.
- Proposed AgeAccel as a cohort-referenced metric of systemic molecular burden.

## Supplementary Material

Supplementary_Materials_bbag605

## Data Availability

This study did not generate new datasets. All datasets analyzed in this study are publicly available, with accession numbers provided in the manuscript and Supplementary Tables. The raw data can be downloaded from the GEO database. The source code, general preprocessing scripts, processed data, trained model and weights, and other resources required for reproducibility are available at https://github.com/YANGMTSYMQ/BAP.
