# Supplementary material for: A blood aging clock defines disease-induced deviation from healthy molecular aging
Source: Brief Bioinform. 2026 Oct 30;27(5):bbag605. doi: 10.1093/bib/bbag605 (PMC13720740; doi:10.1093/bib/bbag605)
Supplement: Supplementary file 1 [file supplementary_materials_bbag605.zip › Supplementary_Materials_bbag605/Supplyinformation_R1_proof_bbag605.docx]

**Supplementary information to：**

**A Blood Aging Clock Defines Disease-Induced Deviation from Healthy Molecular Aging**

Authors: Haotian Li^1^, Sai Liu^1^, Zheng Zhou^3^, Qiulun Lu^1, *^, Guangji Wang^1, *^, Zheying Zhu^2, *^, Xinuo Li^1, *^

^1^State Key Laboratory of Natural Medicines, China Pharmaceutical University, Nanjing 211198, China.

^2^School of Pharmacy, The University of Nottingham, Nottingham NG7 2RD, UK.

^3^Department of Computer Science, RWTH Aachen University, Aachen 52074, Germany.

^*^Email: [xinuo.li@cpu.edu.cn (X.L.)](mailto:xinuo.li@cpu.edu.cn%20(X.L.)); zheying.zhu@nottingham.ac.uk (Z.Z.); [guangjiwang@hotmail.com](mailto:guangjiwang@hotmail.com) (G.W.); [qiulunlu@cpu.edu.cn](mailto:qiulunlu@cpu.edu.cn) (Q.L.)

**Supplementary Figure S1. Validation of shared differentially expressed genes.**

**(A) Expression trajectories of 10 key genes significantly altered in both aging and COVID-19.**

**(B) Boxplots showing age-adjusted expression differences of the 10 genes between COVID-19 samples and healthy controls. Statistical significance was assessed using a two-sided Wilcoxon rank-sum test.**

**Alt text: Expression patterns of 10 genes shared between aging and COVID-19, showing their age-related trajectories and age-adjusted expression differences in COVID-19 samples.**

**Supplementary Figure S2. Overview of external test cohorts and data preprocessing.**

**(A) Density plots showing age distributions across the nine external test cohorts.**

**(B) Bar plots showing sample sizes across the nine external test cohorts.**

**(C) PCA of the nine external test cohorts after normalization and batch-effect correction.**

**(D) Workflow of dataset partitioning and sample selection during BAP model construction.**

**Alt text: Overview of the nine external test cohorts, including their age distributions, sample sizes, data integration after preprocessing, and the dataset partitioning strategy used for BAP development.**

 **Supplementary Figure S3. Analysis of core age-associated features.**

**(A) Venn diagram showing the overlap between the 398 core genes and significant age-associated genes reported by Peters et al.**

**(B) Elbow plot used to determine the optimal number of clusters for Mfuzz analysis.**

**(C) Age-related expression trajectories of the three gene clusters identified by Mfuzz.**

**(D) Interaction networks of the three gene clusters showing hub genes and their connections.**

**(E) Gene Ontology (GO) enrichment analysis of the three gene clusters. Color intensity represents the P value, and circle size represents the number of enriched genes.**

**(F) Scatter plots showing associations between chronological age and inferred immune cell proportions estimated using quanTIseq. Curves indicate fitted trends.**

**Alt text: Analysis of core age-associated features, including overlap with previously reported aging genes, Mfuzz clustering, interaction networks, functional enrichment, and age-related changes in inferred immune cell proportions.**

**Supplementary Figure S4. Model optimization, ensemble construction, and gene-only model validation.**

**(A) Comparison of predictive performance across conventional machine-learning and deep-learning models in different datasets.**

**(B) Optimization of dropout and learning-rate parameters for the DNN model. Circles represent absolute prediction error (lower values indicate better performance), and triangles represent the composite evaluation score (higher values indicate better performance).**

**(C) Performance of the DNN model across ten repeated rounds of 10-fold cross-validation. The top 15 models were selected based on their cross-validation performance and subsequently combined using the external tuning cohort for ensemble optimization.**

**(D–F) Scatter plots showing the predictive performance of the gene-only model trained exclusively on gene-expression features. Panels D, E, and F show performance in the internal test set, external tuning cohort, and independent external test cohort, respectively.**

**(G) Heatmap summarizing the performance of the gene-only model across nine independent external test cohorts using RMSE, MAE, MAD, R², and Spearman correlation as evaluation metrics.** Red indicates better overall performance, whereas blue indicates poorer performance.

**Alt text: Figure showing model comparison and optimization of BAP, together with the predictive performance of a gene-only model across internal and external datasets.**

**Supplementary Figure S5. Performance of the Peters et al. age-prediction model in the BAP external test cohorts.**

**(A) Scatter plots showing the age-prediction performance of the model developed by Peters et al. across the nine independent external test cohorts. The diagonal line represents the ideal prediction line (y = x).**

**(B) Heatmap summarizing the predictive performance of the Peters et al. model across the nine independent external test cohorts using multiple evaluation metrics.**

**Alt text: Performance of the Peters et al. age-prediction model across the same nine independent external test cohorts used to evaluate BAP, showing predicted versus chronological age and cross-cohort performance metrics.**

 **Supplementary Figure S6. Age-dependent bias correction and biological characterization of COVID-19-associated AgeAccel.**

**(A) Scatter plot showing age predictions for COVID-19 patients in the external COVID-19 cohort. The curve represents the age-dependent prediction-bias curve fitted to healthy samples and used for CFA/CSA classification. Red and green points indicate CFA and CSA samples, respectively.**

**(B) Scatter plot showing the corrected prediction deviations of COVID-19 patients after adjustment using the healthy-sample-derived age-dependent bias curve. The line indicates the fitted trend across all COVID-19 samples.**

**(C) Venn diagram showing the overlap among the 398 core genes, immune-related gene sets, and COVID-19 severity-associated gene sets.**

**(D) Scatter plots showing associations between continuous AgeAccel and immune- and COVID-19-related molecular signatures, including cytokine production, interferon response, immune response, neutrophil activation, and the ViP signature. Lines indicate fitted trends.**

**(E) Venn diagram showing the overlap between the 398 core genes and aging-related gene sets.**

**Alt text: Analysis of COVID-19-associated AgeAccel showing age-dependent bias correction for CFA/CSA classification, overlap of core genes with immune-, COVID-19-, and aging-related gene sets, and associations between continuous AgeAccel and immune and inflammatory signatures.**

**Supplementary Figure S7. Association of COVID-19-associated AgeAccel with aging-related molecular signatures.**

**(A) Boxplots showing differences in ssGSEA scores for aging-related signatures between the CFA and CSA groups, including cellular senescence, DNA damage response, and other aging-related pathways. P values calculated using a two-sided Wilcoxon rank-sum test are indicated in the plots.**

**(B) Scatter plots showing associations between continuous AgeAccel and aging-related signatures, including cellular senescence, DNA damage response, and other aging-related pathways. Lines indicate fitted trends.**

**Alt text: Aging-related molecular signatures associated with COVID-19 AgeAccel, showing differences between CFA and CSA groups and associations with continuous AgeAccel.**

**Supplementary Figure S8. Longitudinal dynamics of COVID-19-associated AgeAccel and related molecular signatures.**

**(A–D) Violin plots showing AgeAccel at different time points in the longitudinal COVID-19 cohort (Pre, First, Mid, and Post). Blue indicates the healthy state, and red indicates the COVID-19 state.**

**(E-F)** Longitudinal changes in mean AgeAccel across different stages of COVID-19 progression and recovery, with 95% confidence intervals.

**(G) Comparison of ssGSEA scores for five COVID-19 severity- and inflammation-related gene sets across disease stages. P values calculated using a two-sided Wilcoxon rank-sum test are indicated in the plots.**

**(H) Boxplots showing changes in aging-related functional signatures across different disease stages. Blue indicates the healthy state, and red indicates the COVID-19 state. Statistical significance was assessed using a two-sided Wilcoxon rank-sum test.**

**Alt text: Longitudinal analysis of COVID-19 showing changes in AgeAccel and associated molecular signatures across disease progression and recovery, including COVID-19 severity-, inflammation-, and aging-related pathways.**

**Supplementary Figure S9. AgeAccel and associated molecular signatures across sepsis, influenza, and major depressive disorder.**

**(A) Scatter plot showing BAP-predicted biological age versus chronological age in the sepsis cohort.**

**(B) Violin plot comparing AgeAccel between patients with sepsis and matched healthy controls.**

**(C) Scatter plot showing BAP-predicted biological age versus chronological age in the influenza cohort.**

**(D) Violin plot comparing AgeAccel between patients with influenza and matched healthy controls.**

**(E) Scatter plot showing BAP-predicted biological age versus chronological age in the major depressive disorder (MDD) cohort.**

**(F) Violin plot comparing AgeAccel between patients with MDD and matched healthy controls.**

**(G) Boxplots comparing ssGSEA scores for twelve immune-inflammatory and aging-related gene sets between the sepsis slow-aging (SSA) and sepsis fast-aging (SFA) groups. P values calculated using a two-sided Wilcoxon rank-sum test are indicated in the plots.**

**(H) Scatter plots showing associations between continuous AgeAccel and ssGSEA scores for immune-inflammatory and aging-related gene sets in the sepsis cohort. Lines indicate fitted trends.**

**(I) Boxplots comparing ssGSEA scores for twelve immune-inflammatory and aging-related gene sets between the influenza slow-aging (ISA) and influenza fast-aging (IFA) groups. P values calculated using a two-sided Wilcoxon rank-sum test are indicated in the plots.**

**(J) Scatter plots showing associations between continuous AgeAccel and ssGSEA scores for immune-inflammatory and aging-related gene sets in the influenza cohort. Lines indicate fitted trends.**

**(K) Boxplots comparing ssGSEA scores for twelve immune-inflammatory and aging-related gene sets between the MDD slow-aging (MSA) and MDD fast-aging (MFA) groups. P values calculated using a two-sided Wilcoxon rank-sum test are indicated in the plots.**

**(L) Scatter plots showing associations between continuous AgeAccel and ssGSEA scores for immune-inflammatory and aging-related gene sets in the MDD cohort. Lines indicate fitted trends.**

**Alt text: Multi-disease analysis of AgeAccel in sepsis, influenza, and major depressive disorder, showing biological-age predictions, disease-associated AgeAccel, differences in immune-inflammatory and aging-related signatures between fast- and slow-aging groups, and their associations with continuous AgeAccel.**

**Response Figure 1. Violin plots showing age-prediction distributions across different age groups in the same COVID-19 cohort, based on a gene-only model trained and evaluated using the same strategy as BAP. AgeAccel values are indicated in the plots.**

 **Response Figure 2. Violin plots showing age-prediction distributions across different groups in the same longitudinal COVID-19 cohort, based on a gene-only model trained and evaluated using the same strategy as BAP. AgeAccel values are indicated in the plots.**
